# Enhanced enfortumab vedotin sensitivity in a cisplatin-resistant bladder cancer model

**DOI:** 10.1371/journal.pone.0361593

**Published:** 2026-10-29

**Authors:** Taisuke Tobe, Jun Teishima, Takuto Hara, Yusuke Shiraishi, Hideto Ueki, Naoto Wakita, Yasuyoshi Okamura, Yukari Bando, Kotaro Suzuki, Tomoaki Terakawa, Akihisa Yao, Koji Chiba, Hideaki Miyake

**Affiliations:** Division of Urology, Department of Surgery Related, Kobe University Graduate School of Medicine, Japan; Icahn School of Medicine at Mount Sinai Department of Pharmacological Sciences, UNITED STATES OF AMERICA

## Abstract

**Objective:**

Enfortumab vedotin (EV) and cisplatin (CDDP) are central treatments for advanced urothelial carcinoma, yet the influence of prior CDDP exposure on subsequent EV efficacy remains unclear. In this report, we characterized EV-related drug responses in a CDDP-resistant bladder cancer subline.

**Methods:**

A CDDP-resistant subline (HT1376-CR) was generated from parental bladder cancer cell (HT1376/P) through continuous exposure to CDDP. Sensitivities to CDDP, EV, and the EV payload monomethyl auristatin E (MMAE) were compared between HT1376-CR and HT1376/P. Nectin-4 expression, EV binding, and drug-resistance-related gene expression were evaluated. The effect of ABCB1 silencing on drug response was also examined. In vivo EV responses were explored using xenograft models.

**Results:**

HT1376-CR cells showed increased sensitivity to EV in vitro and in vivo. HT1376-CR cells were more sensitive to MMAE and showed greater G2/M accumulation after MMAE exposure. In contrast, cell-surface nectin-4 expression and EV binding were reduced. ABCB1 was markedly upregulated in HT1376-CR, and ABCB1 silencing increased sensitivity to CDDP, MMAE, and EV within the resistant subline.

**Conclusion:**

The CDDP-resistant HT1376 subline showed enhanced sensitivity to EV, and ABCB1 suppression further increased EV sensitivity within this resistant subline.

## Introduction

Bladder cancer is among the most common malignancies worldwide, with approximately 430,000 new cases diagnosed annually, ranking as the 11^th^ most frequent cancer globally [1]. Although treatment options have expanded in recent years, the prognosis for patients with advanced bladder cancer remains poor, highlighting the urgent need for more effective systemic therapies.

For patients with locally advanced or metastatic urothelial carcinoma, systemic therapy has been the cornerstone of disease management. Historically, cisplatin (CDDP)-based combination chemotherapy has served as the standard of care. In recent years, however, the therapeutic landscape has evolved dramatically with the advent of immune checkpoint inhibitors (ICIs) and antibody–drug conjugates (ADCs). Among the latter, enfortumab vedotin (EV) has emerged as a pivotal therapeutic agent. EV exerts its antitumor effect by binding to nectin-4, followed by internalization into tumor cells, where its cytotoxic payload, monomethyl auristatin E (MMAE), disrupts microtubule dynamics [2]. EV is now used either in combination with pembrolizumab as a first-line regimen or as a monotherapy following platinum-based therapy [3,4]. Despite these advances, platinum-based chemotherapy continues to play a critical role, particularly in regimens such as CDDP plus gemcitabine combined with nivolumab, and remains indispensable for patients ineligible for ICIs [5]. Consequently, the current therapeutic paradigm often relies on platinum-based chemotherapy followed by EV as a subsequent-line therapy.

However, the impact of prior exposure to platinum on subsequent EV efficacy remains unresolved. Reduced nectin-4 expression has been reported in cisplatin-resistant cells [6], and in breast cancer models, ABC transporter B family member 1 (ABCB1, P-glycoprotein/multidrug resistance 1) upregulation has been implicated in resistance to nectin-4-targeted ADCs [7]. These observations raise the possibility that acquired CDDP resistance may be accompanied by changes relevant to EV response.

In this study, we established a CDDP-resistant bladder cancer subline and compared the sensitivities of the parental and CDDP-resistant cell lines to CDDP, EV, and MMAE. We also characterized nectin-4 expression, EV binding, and ABC transporter expression associated with the acquisition of cisplatin resistance.

## Materials and Methods

### Cell culturing

The human bladder cancer cell line HT1376 (ATCC, CRL-1472) was cultured in Eagle's Minimum Essential Medium (FUJIFILM Wako Pure Chemical Corporation, 051–076615) supplemented with 10% fetal bovine serum (BIOSERA, FB-1003/500) at 37 °C in a humidified atmosphere of 5% CO_2_. Stable CDDP-resistant HT1376 cell lines were established by stepwise exposure to increasing concentrations of CDDP (FUJIFILM Wako Pure Chemical Corporation, 039–20093), starting at 0.01 µg/mL and progressively increasing at each passage until a final resistance concentration of 1.5 µg/mL was reached.

### Cell viability assay

Cells (1.0 × 10³ per well) were seeded into 96-well plates and treated with CDDP, EV (Selleck, D4053), or MMAE (Selleck, S7721). Cell viability was assessed using the Cell Counting Kit-8 (CCK-8) reagent (Dojindo, CK04) 3 days after CDDP or MMAE treatment, and 5 days after EV treatment. Plates were incubated with CCK-8 reagent for 2 h in the dark, and the absorbance was measured at 450 nm using a Varioskan LUX microplate reader (Thermo Fisher Scientific). IC_50_ values were determined by nonlinear regression using a four-parameter logistic model in GraphPad Prism (version 10.5.0). Data are presented as the standard error of the mean (SEM) from three independent experiments (*n* = 3).

### Cell proliferation

Cells (1 × 10³ per well) were seeded into 96-well culture plates in 100 µl of medium and cultured for 1, 2, 3, 4, or 5 days. Cell proliferation was quantified using CCK-8, as described above. Absorbance values at each time point were normalized to the day‑1 measurements for the corresponding conditions to yield relative proliferation. Data are presented as the mean ± SEM from three independent experiments (*n* = 3).

### Western blotting

Cells were washed in PBS and lysed with RIPA buffer (FUJIFILM Wako Pure Chemical Corporation, 182−02451) supplemented with a protease inhibitor cocktail (Roche, 05892791001). Protein concentrations were determined using the BCA Protein Assay kit (Thermo Fisher, 23225). Lysates were mixed with 4× Laemmli Sample Buffer (Bio-Rad, #1610747) containing 2-mercaptoethanol (Sigma-Aldrich, M3148) and boiled at 95 °C for 5 min. Proteins were separated by SDS-PAGE and transferred to PVDF membranes (Merck, IPVH00010). Membranes were blocked with 5% skim milk in PBS-T (0.05% Tween 20) and incubated overnight with the primary antibodies: anti-nectin-4 (Abcam, ab235897), anti-ABCB1 (Cell Signaling Technology, E1Y7S), and anti-β-actin (Abcam, ab227387). The following day, membranes were washed with PBS-T and incubated with a horseradish peroxidase-conjugated secondary antibody (Abcam, ab7090) for 1 h at room temperature. Protein bands were visualized using an enhanced chemiluminescence detection reagent (Cytiva, RPN2232) and quantified using an Amersham Imager 600 system (Cytiva). Data are presented as the mean ± SEM from three independent experiments (*n* = 3).

### Flow Cytometry

HT1376/P and HT1376-CR cells were seeded in culture plates and detached using enzyme-free cell dissociation solution (Sigma-Aldrich, C5789). To assess cell surface nectin-4 expression, cells were incubated with an anti-nectin-4 antibody (R&D systems, FAB2659P) or isotype control antibody (R&D systems, IC0041P) for 30 min at 4 °C. Flow cytometric analysis was performed using a CytoFLEX flow cytometer (Beckman Coulter), and mean fluorescence intensity (MFI) was quantified using FlowJo software (BD Biosciences). Relative nectin-4 expression was determined by subtracting the MFI of the control antibody from that of the anti-nectin-4 antibody.

To evaluate the binding of EV to the cell surface, cells were incubated with EV (3.3 or 13.2 nM) for 30 min at 4 °C, followed by incubation with APC anti-human IgG Fc antibody (BioLegend, 410711) for an additional 30 min at 4°C. Samples were analyzed using the same procedure described above.

### Quantitative real-time PCR

Total RNA was extracted from cell lines using TRIzol reagent (Invitrogen, 15596026) according to the manufacturer’s instructions. The RNA concentration and purity were assessed using a NanoDrop One spectrophotometer (Thermo Fisher Scientific). cDNA was synthesized using a reverse transcription kit (Thermo Fisher Scientific, 11766050) according to the manufacturer’s protocol. Quantitative PCR was performed using SYBR Green Master Mix (TOYOBO, QPX-201). Primer sequences used for PCR are listed in S1 Table. Data are presented as the mean ± SEM from three independent experiments (*n* = 3).

### Cell cycle assay

HT1376/P and HT1376-CR cells were seeded at a density of 2 × 10^5^ cells per well. After 24 h, the cells were exposed to MMAE (0.0 or 2.0 nM) for an additional 24 h. Cells were harvested, stained with Cell Cycle Assay Solution Deep Red Kit (Dojindo, C548), and analyzed by flow cytometry (Beckman Coulter, CytoFLEX). Cell cycle distribution (G1, S, and G2/M phases) was assessed using FlowJo software (BD Biosciences).

### RNA interference

HT1376-CR cells were transfected with ABCB1 siRNA (Thermo Fisher Scientific, s10418 and s10419). A non-targeting siRNA (Thermo Fisher Scientific, 94566) served as the negative control. Cells (2 × 10^5^ per well) were seeded in 6-well plates and transfected the following day using Lipofectamine RNAiMAX Transfection Reagent (Thermo Fisher Scientific, 13778030), according to the manufacturer’s protocol.

### Xenograft tumor growth

Female BALB/c nude mice (8 weeks old, body weight 26–28 g) were subcutaneously injected with HT1376/P or HT1376-CR cells (3 × 10^6^) into the flank. When the longest tumor diameter reached 10 mm, mice were randomly assigned to receive either EV (1 mg/kg) or vehicle control (physiological saline) by intraperitoneal injection once weekly for 3 weeks. Tumor length (the longest diameter) was measured with a caliper. For volume calculation, tumor width was approximated as half of the length (width = length/2), and tumor volumes were calculated using the formula length × width^2^ × π/6. After 21 days of treatment, mice were deeply anesthetized with isoflurane. After confirming death by cessation of respiration, tumors were excised and weighed. Both the length and width of each excised tumor were directly measured, and terminal tumor volume was calculated using the formula length × width² × π/6. Tumors were subsequently fixed in 4% paraformaldehyde. Relative tumor volume (RTV) was calculated for each mouse by dividing the tumor volume on day 22 by the tumor volume on day 1. RTV values are presented as mean ± SEM for each group.

Because death or severe deterioration of animal health could potentially occur as a result of tumor progression or drug treatment, predefined humane endpoints were established prior to initiation of the study. Animals were euthanized at any time during the experimental period if any of the following criteria were observed: severe adverse effects related to drug administration, including marked inflammation or secondary infection; impaired mobility, such as persistent recumbency or crouching posture; or signs of organ or tissue damage, including dyspnea or labored breathing, symptoms of shock, diarrhea or vomiting, seizures, or circling behavior. In addition, animals were euthanized if body weight loss exceeded 20% compared with control animals or if body weight loss of 25% or more was observed within a 7-day period. Euthanasia was performed by overdose of isoflurane anesthesia.

Animal health status and behavior were monitored twice weekly throughout the experimental period, which lasted approximately three months from tumor cell inoculation to study completion. A total of 14 mice were used in this study. All animals were euthanized at the experimental endpoint. None of the animals met the humane endpoint criteria prior to the planned experimental endpoint, and therefore no mice were euthanized prematurely due to adverse treatment effects.

Animal experiments in this study were approved by Kobe University (approval number P-250503). This study was carried out in strict accordance with the recommendations in the Guide for the Care and Use of Laboratory Animals of the National Institutes of Health. All animal procedures were performed by personnel who were appropriately trained and certified in laboratory animal care and handling, in accordance with institutional guidelines.

### Statistical analysis

Continuous variables in experimental data are reported as means with 95% confidence intervals. Group comparisons were performed using Student’s *t*-test for continuous variables. To determine whether the antitumor effect of EV differed between tumor models, RTV values were analyzed by two-way ANOVA with factors for tumor model (HT1376/P vs HT1376-CR), treatment (EV vs vehicle).

For all statistical analyses, *P* < 0.05 was considered statistically significant. In all figures, statistical significance is denoted as follows: ns, ≥ 0.05; *, 0.01 ≤ *P* < 0.05; **, 0.001 ≤ *P*  <   0.01; ***, *P* < 0.001.

## Results

### Establishment of a cisplatin-resistant cell line

A CDDP-resistant subline (HT1376-CR) was established from the parental HT1376 (HT1376/P) bladder cancer cell line by gradually increasing the CDDP concentration during culture. The resulting HT1376-CR cells exhibited reduced sensitivity to CDDP, with a half-maximal inhibitory concentration (IC_50_) more than threefold higher than that of parental HT1376/P cells (Fig 1a). HT1376-CR cells also demonstrated increased proliferative capacity, with a faster growth rate than HT1376/P cells (Fig 1b). The individual experimental data underlying Figs 1–5 are provided in S1 File.

**Fig 1:**
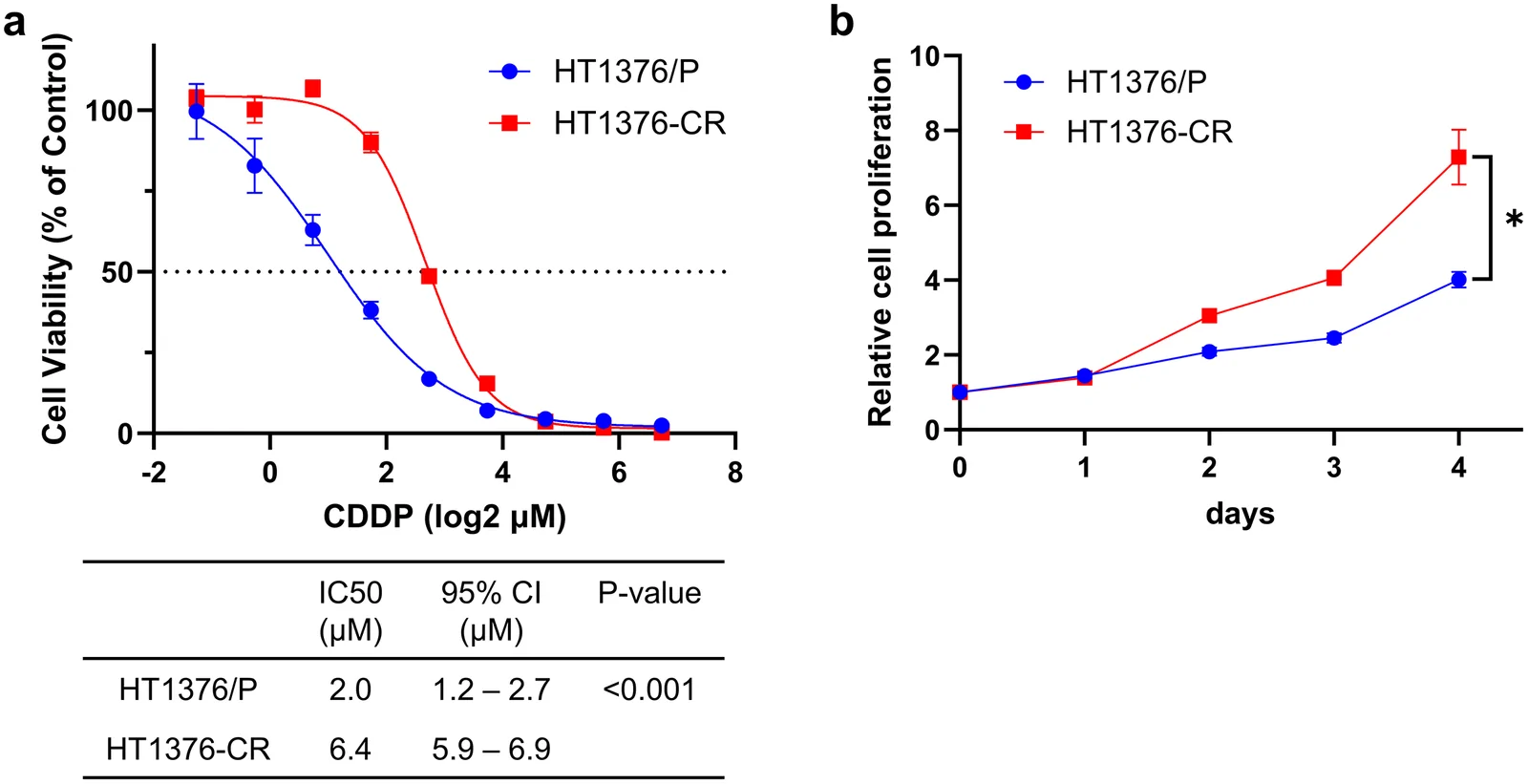
Comparison of CDDP sensitivity and proliferation between HT1376/P and HT1376-CR. (a) Cell viability of HT1376/P and HT1376-CR cells following CDDP treatment. The x-axis represents the CDDP concentration, and the y-axis represents the relative cell number normalized to the cell number at 0 nM. (b) Proliferation growth curves of HT1376/P and HT1376-CR cells. Values were normalized to the cell number at day 0 for each group. Statistical significance is denoted as follows: *, 0.01 ≤ P  <   0.05.

**Fig 2:**
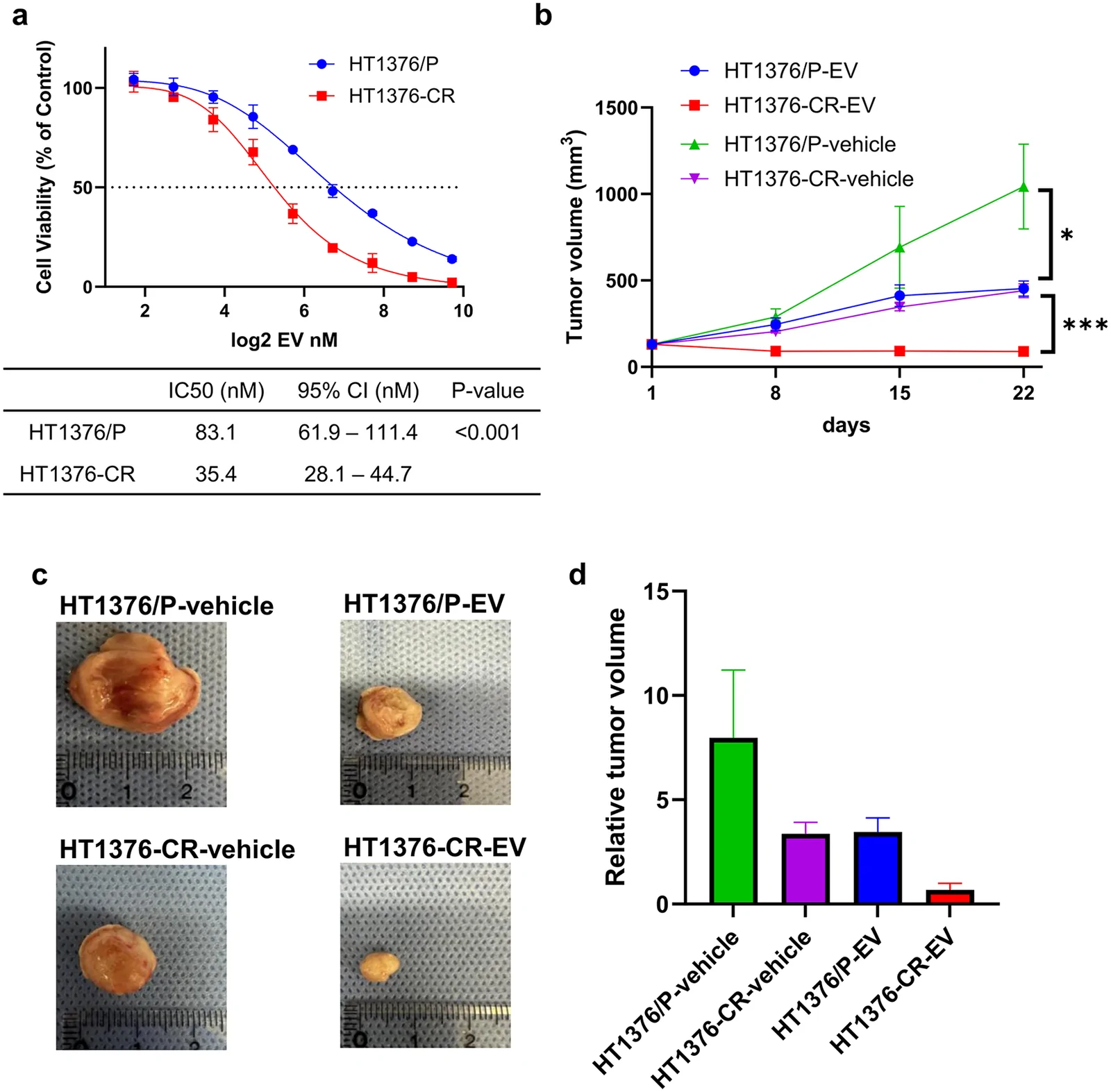
Comparison of EV sensitivity between HT1376/P and HT1376-CR. (a) Cell viability of HT1376/P and HT1376-CR cells in response to EV treatment. The x-axis represents the drug concentration, and the y-axis indicates the relative cell number after exposure to the indicated drug concentrations. Values are normalized to the cell number at 0 nM. (b) Mean tumor volumes of HT1376/P xenografts treated with EV (HT1376/P-EV) or vehicle (HT1376/P-vehicle) and HT1376-CR xenografts treated with EV (HT1376-CR-EV) or vehicle (HT1376-CR-vehicle) at the indicated time points during EV treatment. (c) Representative macroscopic appearance of xenograft tumors after 21 days of EV treatment. (d) Relative tumor volume at day 22, calculated by normalizing tumor volume to baseline. Statistical significance is denoted as follows: *, 0.01 ≤ P < 0.05; ***, P < 0.001.

**Fig 3:**
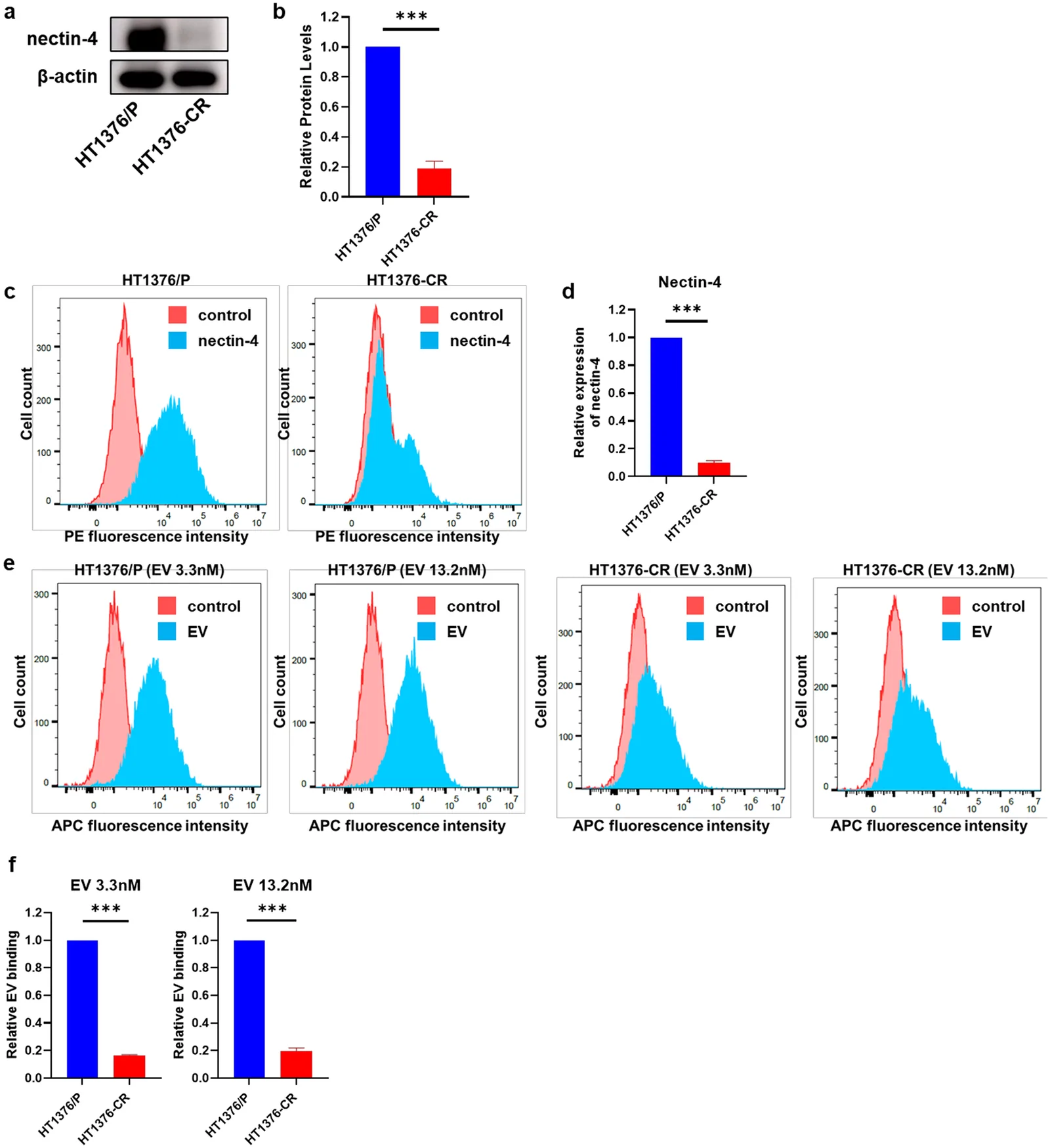
Comparison of nectin-4 expression and EV binding between HT1376/P and HT1376-CR. (a) Immunoblot analysis of nectin-4 expression in HT1376/P and HT1376-CR cells. β-actin was used as the loading control. (b) Quantification of relative nectin-4 protein levels by immunoblot analysis. (c) Representative flow cytometry histograms showing cell-surface nectin-4 expression in HT1376/P and HT1376-CR cells. (d) Quantification of relative cell-surface nectin-4 expression measured by flow cytometry. (e) Representative flow cytometry histograms showing EV binding to HT1376/P and HT1376-CR cells. (f) Quantification of relative EV binding measured by flow cytometry. Statistical significance is denoted as follows: ***, P < 0.001. The original immunoblot images are provided in S2 File.

**Fig 4:**
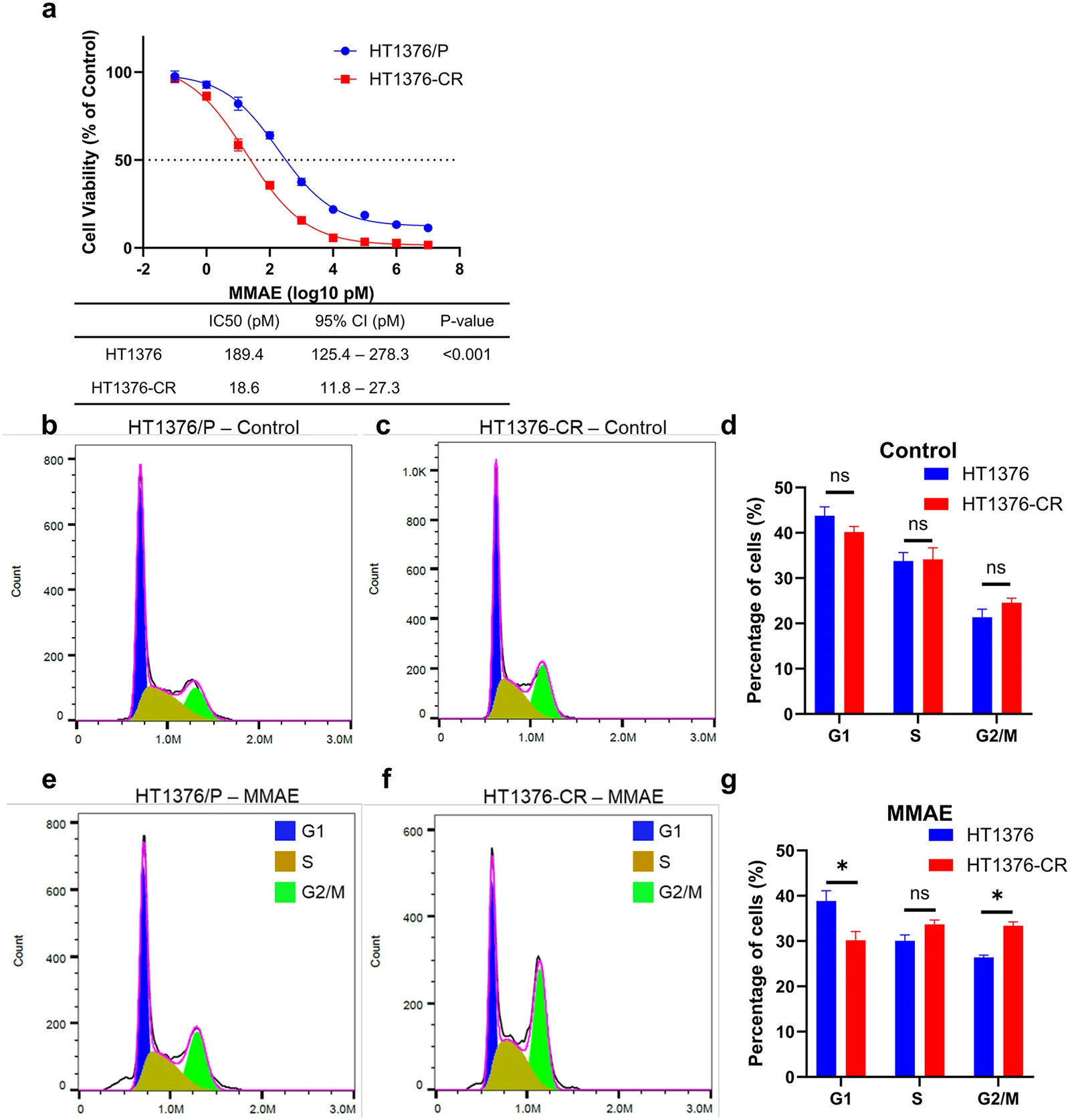
Comparison of sensitivity to MMAE and cell cycle distribution between HT1376/P and HT1376-CR. (a) Cell viability of HT1376/P and HT1376-CR cells following MMAE treatment. The x-axis represents the concentration of MMAE, whereas the y-axis represents the relative cell viability 3 days after treatment, normalized to the value at 0 nM. (b, e) Representative histograms of cell cycle analysis for HT1376/P cells treated with MMAE at 0 nM (b) and 2 nM (e). (d) Quantification of the cell cycle distribution in HT1376/P and HT1376-CR cells treated without MMAE. (c, f) Representative histograms of cell cycle analysis in HT1376-CR cells treated with MMAE at 0 nM (c) and 2 nM (f). (g) Quantification of cell cycle distribution in HT1376-CR and HT1376-CR cells treated with MMAE at 2 nM. Statistical significance is denoted as follows: ns, ≥ 0.05; *, 0.01 ≤ P  <   0.05.

**Fig 5:**
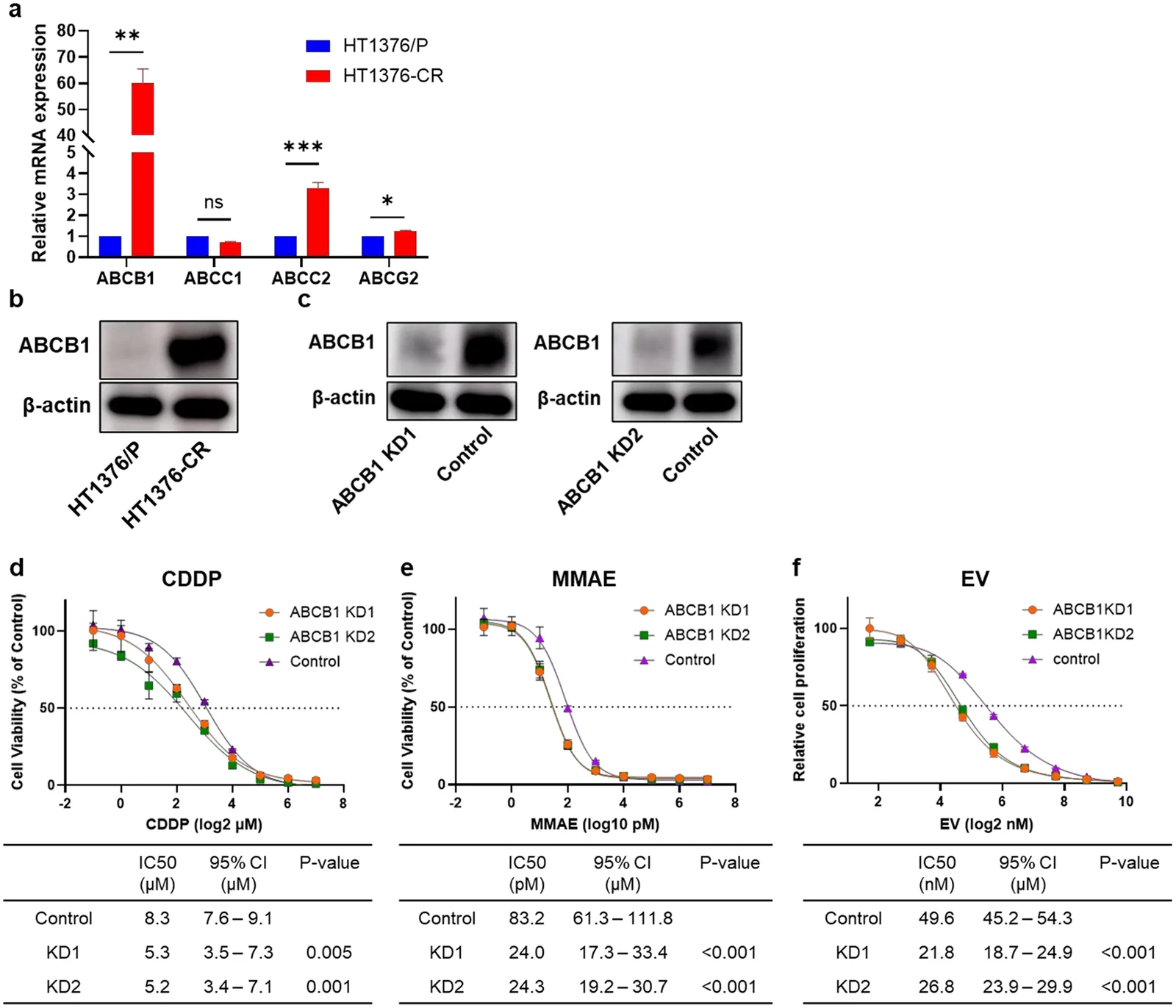
Comparison of ABC transporter expression and the impact of ABCB1 knockdown on drug sensitivity in HT1376-CR. (a) Quantitative PCR analysis of ABC transporter isoform expression in HT1376/P and HT1376-CR cells. Expression levels were normalized to β-actin and are presented as fold change relative to HT1376/P cells. (b) Immunoblot analysis of ABCB1 expression in HT1376/P and HT1376-CR cells. β-actin was used as the loading control. (c) Immunoblot analysis of ABCB1 expression in HT1376-CR with ABCB1 knockdown (KD1/2) and control. (d–f) Cell viability of ABCB1-knockdown and control HT1376-CR cells following treatment with CDDP (d), MMAE (e), EV (f). Statistical significance is denoted as follows: ns, ≥ 0.05; *, 0.01 ≤ P  <   0.05; **, 0.001 ≤ P  <   0.01; ***, P < 0.001. The original immunoblot images are provided in S2 File.

### Comparison of enfortumab vedotin sensitivity between parental and cisplatin-resistant bladder cancer cell lines

EV sensitivity was evaluated in HT1376/P cells and their CDDP-resistant counterparts. The CDDP-resistant cells exhibited markedly enhanced sensitivity to EV compared with the parental cells, showing lower viability across the tested concentration range (Fig 2a). We next examined whether this difference in EV sensitivity was also observed in vivo using a mouse xenograft model. Tumor growth curves demonstrated that EV treatment suppressed tumor growth in both HT1376/P and HT1376-CR xenografts (Fig 2b, c).

To quantitatively compare treatment responses between tumor models, RTV was calculated by normalizing tumor volume to baseline (Fig 2d). Two-way ANOVA of log-transformed RTV values at day 22 revealed a significant interaction between tumor model and treatment (p = 0.039), indicating that the antitumor efficacy of EV differed between HT1376/P and HT1376-CR tumors. As a supportive analysis, terminal tumor volumes were calculated from the directly measured length and width of the excised tumors. This analysis yielded a consistent result, with a significant tumor model-by-treatment interaction (p = 0.0035).

### Comparison of nectin-4 expression and EV binding between parental and cisplatin-resistant bladder cancer cell lines

EV exerts its cytotoxic effects by binding to nectin-4, followed by internalization, and previous studies report that nectin-4 expression levels affect EV efficacy [6,8]. Accordingly, we quantified nectin-4 expression in both the parental cell line and its CDDP-resistant derivative. Nectin-4 protein levels were markedly reduced in the CDDP-resistant line compared with the parental line (Fig 3a, b).

To further assess cell-surface nectin-4 expression, flow cytometric analysis was performed. Consistent with the immunoblot findings, cell-surface nectin-4 expression was significantly lower in HT1376-CR cells than in HT1376/P cells (Fig. 3c, d).

We next examined EV binding to the cell surface. HT1376/P and HT1376-CR cells were incubated with EV (3.3 or 13.2 nM), and EV binding was quantified by flow cytometry. EV binding was reduced in HT1376-CR cells compared with HT1376/P cells at both concentrations tested (Fig. 3e, f).

### Comparison of sensitivity to MMAE between parental and cisplatin-resistant cell lines

The antitumor activity of EV is mediated by its cytotoxic payload, MMAE. The enhanced EV sensitivity observed in the CDDP-resistant line prompted an assessment of MMAE responsiveness directly. HT1376-CR cells showed greater sensitivity to MMAE than the parental cells (Fig 4a). MMAE is a microtubule inhibitor that induces G2/M arrest. Thus, we examined whether baseline or drug-induced cell cycle distributions differed between the two cell lines. Under untreated conditions, the proportions of cells in the G1, S, and G2/M phases were similar between HT1376/P and HT1376-CR cell lines (Fig 4b–d). In contrast, exposure to 2.0 nM MMAE caused a marked increase in the G2/M fraction in HT1376-CR relative to HT1376/P, indicating a stronger G2/M arrest in the CDDP-resistant cells (Fig 4e–g).

### Comparison of ABC transporter expression levels between parental and cisplatin-resistant cell lines

Both CDDP and MMAE can be excreted extracellularly through ABC transporters, prompting an evaluation of whether efflux-related changes contribute to the CDDP-resistant cell line phenotype [9]. Among the transporters examined, ABCB1 expression was markedly upregulated in the CDDP-resistant subline (Fig 5a, b). Initially, we investigated whether inhibition of ABCB1 restores CDDP sensitivity in the resistant cells. ABCB1 was efficiently knocked down by siRNA in HT1376-CR cells (Fig 5c), and these cells showed greater sensitivity to CDDP than the control siRNA group (Fig 5d). We next investigated the impact of ABCB1 knockdown on responses to MMAE and EV. ABCB1-depleted HT1376-CR cells displayed a significant increase in sensitivity to MMAE and EV relative to controls (Fig 5e, 5f). These findings indicate that elevated ABCB1 expression in HT1376-CR contributes to reduced responsiveness to CDDP, MMAE, and EV.

## Discussion

In this study, the CDDP-resistant HT1376 subline showed greater sensitivity to EV than its parental counterpart. These findings suggest that cellular changes associated with the acquisition of cisplatin resistance may influence EV responsiveness. Although the clinical relevance of this observation remains unclear, the present findings support further investigations comparing outcomes between patients receiving EV upfront and those treated with EV after extensive platinum-based chemotherapy.

In the present study, EV sensitivity was enhanced in the cisplatin-resistant subline. Although reduced nectin-4 expression has been associated with attenuated EV efficacy in previous reports [6,8], EV sensitivity in this subline was increased rather than decreased. This finding suggests that the expression level of the ADC target antigen does not necessarily correlate with its efficacy. This apparent paradox was not resolved by the present data; future studies should determine which step of the EV mechanism of action, such as target binding, internalization, trafficking, or payload release, underlies the enhanced response.

Cisplatin-resistant cells exhibited increased sensitivity to MMAE, the cytotoxic payload of EV. MMAE disrupts microtubule dynamics and induces mitotic arrest [10], and its efficacy can depend on the proliferative status of tumor cells [11]. HT1376-CR cells also exhibited a higher proliferation rate than parental cells and showed more pronounced G2/M arrest following MMAE exposure. Some biological alterations acquired during the development of cisplatin resistance may be associated with the enhanced susceptibility to MMAE, and further studies will be required to clarify the molecular basis underlying this phenotype.

We also identified a marked upregulation of the drug efflux transporter ABCB1 in HT1376-CR cells. ABC transporters are proteins that use adenosine triphosphate to export various substrates, including MMAE [12,13], and confer multidrug resistance by exporting anticancer agents from cells. Several studies have reported increased ABCB1 expression in CDDP-resistant bladder cancer cell lines [14,15]. Additionally, increased ABCB1 expression has been associated with reduced efficacy of MMAE-based ADCs, including EV [7,16]. Consistent with these reports, silencing ABCB1 restored sensitivity to CDDP and further increased the efficacy of EV and MMAE in our model. These findings support a functional role for ABCB1-mediated drug efflux in modulating the responses to CDDP, MMAE, and EV. In addition to ABCB1, ABCC2 expression was also significantly increased in HT1376-CR cells. ABCC2 has been reported to mediate the efflux of cisplatin–glutathione conjugates and is considered one of the mechanisms underlying acquired cisplatin resistance [9]. Although the functional role of ABCC2 was not directly investigated in the present study, its upregulation may contribute to the cisplatin-resistant phenotype of HT1376-CR cells.

This study has several limitations. First, the findings were derived from a single cisplatin-resistant subline established from one bladder cancer cell line. Therefore, the findings cannot be generalized to cisplatin-resistant bladder cancer, and clonal selection during prolonged culture cannot be excluded as a contributor to the observed phenotype. Validation using independently established resistant sublines and additional bladder cancer models is required. Second, tumor volume in the xenograft experiments was calculated using an estimated width (width = length/2) rather than direct bidimensional caliper measurements. This approach assumes a fixed length-to-width ratio and may introduce measurement error if the actual tumor shape differs from this assumption. This limitation should be considered when interpreting the in vivo comparisons between the two models.

## Conclusions

Our results showed that the CDDP-resistant HT1376 subline exhibited greater sensitivity to both EV and MMAE than its parental counterpart. ABCB1 silencing further increased EV sensitivity within the resistant subline. These findings represent observations from a single resistant model and require validation in independent models.

## Supporting information

S1 Table Primer sequences used for polymerase chain reaction. (PDF)

S1 File Dataset supporting the experimental results of this study. (PDF)

S2 File Raw Western Blot and gel images. (PDF)
