# Supplementary material for: Enhanced enfortumab vedotin sensitivity in a cisplatin-resistant bladder cancer model
Source: PLoS One. 2026 Oct 29;21(10):e0361593. doi: 10.1371/journal.pone.0361593 (PMC13718962; doi:10.1371/journal.pone.0361593)
Supplement: Supplementary file 1 [file pone.0361593.s001.pdf]

S1 Table. Primer sequences used for polymerase chain reaction

Gene name	sequence	
nectin-4	Forward	5’-CAAAATCTGTGGCACATTGG-3’
	Reverse	5’-GCTGACATGGCAGACGTAGA-3’
ABCB1	Forward	5'-GCTGTCAAGGAAGCCAATGCCT-3'
	Reverse	5'-TGCAATGGCGATCCTCTGCTTC-3’
ABCC1	Forward	5'-CCGTGTACTCCAACGCTGACAT-3’
	Reverse	5'-ATGCTGTGCGTGACCAAGATCC-3’
ABCC2	Forward	5'-GCCAACTTGTGGCTGTGATAGG-3’
	Reverse	5'-ATCCAGGACTGCTGTGGGACAT-3’
ABCG2	Forward	5'-GTTCTCAGCAGCTCTTCGGCTT-3’
	Reverse	5'-TCCTCCAGACACACCACGGATA-3’
β-actin	Forward	5’-GGCCACGGCTGCTTC-3’
	Reverse	5’-GTTGGCGTACAGGTCTTTGC-3’
