# Supplementary material for: Enhanced enfortumab vedotin sensitivity in a cisplatin-resistant bladder cancer model
Source: PLoS One. 2026 Oct 29;21(10):e0361593. doi: 10.1371/journal.pone.0361593 (PMC13718962; doi:10.1371/journal.pone.0361593)
Supplement: Supplementary file 2 [file pone.0361593.s002.pdf]

Fig. 1

a

The relative cell number normalized to the cell number at 0 nM.

		X	Group A			Group B		
		CDDP (log2 µM)	HT1376			HT1376-CR		
	×	X	A:Y1	A:Y2	A:Y3	B:Y1	B:Y2	B:Y3
1	Title	6.74	2.529900	1.895500	3.015600	0.350600	0.090500	0.172000
2	Title	5.74	5.044500	4.048200	2.504000	2.734500	1.228500	1.400500
3	Title	4.74	4.661100	4.752200	3.809900	4.277100	3.103600	3.685500
4	Title	3.74	7.942300	7.189300	5.883100	14.962800	13.410100	17.899300
5	Title	2.74	17.387300	17.194700	15.966600	48.899200	46.825300	50.098300
6	Title	1.74	37.948500	33.698900	42.716700	95.400400	90.055600	84.754300
7	Title	0.74	56.455100	60.235600	72.172900	110.265000	101.900900	108.108100
8	Title	-0.26	66.237400	93.420000	88.799100	94.811400	97.504200	108.353800
9	Title	-1.26	84.789900	99.810500	114.391500	98.878100	107.422700	104.950900

b

Quantitative cell proliferation data normalized to day 0.

		X	Group A			Group B		
		days	HT1376/P			HT1376-CR		
	×	X	A:Y1	A:Y2	A:Y3	B:Y1	B:Y2	B:Y3
1	Title	0.0	1.000000	1.000	1.000000	1.000000	1.000000	1.000000
2	Title	1.0	1.328767	1.595	1.417840	1.406780	1.437500	1.314607
3	Title	2.0	1.904110	2.070	2.281690	2.949153	3.352273	2.825843
4	Title	3.0	2.200913	2.575	2.586854	3.689266	4.335227	4.168539
5	Title	4.0	3.584475	4.235	4.211268	7.858757	8.187500	5.825843

Fig. 2

a

Relative cell viability after EV treatment, normalized to 0 nM.

Table format: XY	X	Group A			Group B		
	log2 EV nM	HT1376/P			HT1376-CR		
	X	A:Y1	A:Y2	A:Y3	B:Y1	B:Y2	B:Y3
1 Title	9.718088	15.87207	14.46085	11.64991	1.731793	3.996593	0.787791
2 Title	8.718088	24.20682	22.47634	21.44086	2.788524	8.561085	3.219129
3 Title	7.718088	39.02698	36.79381	35.14157	7.451642	21.374320	7.040687
4 Title	6.718088	42.53054	53.58474	48.41228	16.715570	20.579260	21.307420
5 Title	5.718088	69.13811	69.98136	67.79571	31.338440	32.299280	46.606320
6 Title	4.718088	75.86105	96.21451	84.48002	68.692720	56.222050	78.371280
7 Title	3.718088	89.49779	99.67737	97.20164	86.036610	72.783420	93.237360
8 Title	2.718088	95.46997	109.39200	96.74517	91.605870	98.537660	95.986900
9 Title	1.718088	110.12890	102.94670	99.68907	95.240820	113.068800	101.106000

b

Individual tumor volumes of HT1376/P and HT1376-CR xenografts treated with EV.

Table format: XY	X	Group A				Group B				Group C				Group D			
	days	HT1376/P-EV				HT1376-CR-EV				HT1376/P-vehicle				HT1376-CR-vehicle			
	X	A:Y1	A:Y2	A:Y3	A:Y4	B:Y1	B:Y2	B:Y3	B:Y4	C:Y1	C:Y2	C:Y3	C:Y4	D:Y1	D:Y2	D:Y3	D:Y4
1 Title	1.0	130.8333	130.8333	130.8333	130.8333	130.83330	130.83330	130.8333	130.83330	130.8333	130.8333	130.8333		130.8333	130.8333	130.8333	
2 Title	8.0	151.4559	274.3774	226.0800	329.1055	69.53020	89.15925	112.1732	92.23344	255.5339	382.5837	231.7792		214.9634	209.5444	188.7790	
3 Title	15.0	359.0067	587.7197	407.1711	294.1252	62.08722	66.98667	151.4559	86.15414	516.0475	1160.4580	398.8618		336.4187	390.6662	314.7986	
4 Title	22.0	546.0043	496.6978	424.1345	343.8394	50.89639	62.08722	130.8333	115.75300	940.5683	1510.2320	677.4180		374.6135	432.7900	516.0475	

d

Relative tumor volume (RTV) of each xenograft at day 22, calculated by normalizing tumor volume to baseline.

	Group A	Group B	Group C	Group D
	HT1376/P-vehicle	HT1376-CR-vehicle	HT1376/P-EV	HT1376-CR-EV
1	7.189059	2.863289	4.173282	0.389017
2	11.543180	3.307950	3.796417	0.474552
3	5.177719	3.944313	3.241793	1.000000
4			2.628073	0.884737

Fig. 2

Individual tumor measurements and body weight data in xenograft experiments										
Mouse ID	Tumor	Treatment	Day	Length (mm)	Tumor volume (mm3)	Tumor weight (g)	Body weight (g)	Excised tumor length (mm)	Excised tumor width (mm)	Exicised tumor volume (mm3)
M1	HT1376/P	Enfortumab Vedotin	1	10.0	130.9		27			
M2	HT1376/P	Enfortumab Vedotin	1	10.0	130.9		27			
M3	HT1376/P	Enfortumab Vedotin	1	10.0	130.9		27			
M4	HT1376/P	Enfortumab Vedotin	1	10.0	130.9		26			
M5	HT1376-CR	Enfortumab Vedotin	1	10.0	130.9		27			
M6	HT1376-CR	Enfortumab Vedotin	1	10.0	130.9		28			
M7	HT1376-CR	Enfortumab Vedotin	1	10.0	130.9		27			
M8	HT1376-CR	Enfortumab Vedotin	1	10.0	130.9		28			
M1	HT1376/P	Enfortumab Vedotin	8	10.5	151.5		27			
M2	HT1376/P	Enfortumab Vedotin	8	12.8	274.5		27			
M3	HT1376/P	Enfortumab Vedotin	8	12.0	226.2		27			
M4	HT1376/P	Enfortumab Vedotin	8	13.6	329.3		27			
M5	HT1376-CR	Enfortumab Vedotin	8	8.1	69.6		27			
M6	HT1376-CR	Enfortumab Vedotin	8	8.8	89.2		27			
M7	HT1376-CR	Enfortumab Vedotin	8	9.5	112.2		27			
M8	HT1376-CR	Enfortumab Vedotin	8	8.9	92.3		27			
M1	HT1376/P	Enfortumab Vedotin	15	14.0	359.2		28			
M2	HT1376/P	Enfortumab Vedotin	15	16.5	588.0		27			
M3	HT1376/P	Enfortumab Vedotin	15	14.6	407.4		28			
M4	HT1376/P	Enfortumab Vedotin	15	13.1	294.3		27			
M5	HT1376-CR	Enfortumab Vedotin	15	7.8	62.1		27			
M6	HT1376-CR	Enfortumab Vedotin	15	8.0	67.0		29			
M7	HT1376-CR	Enfortumab Vedotin	15	10.5	151.5		28			
M8	HT1376-CR	Enfortumab Vedotin	15	8.7	86.2		27			
M1	HT1376/P	Enfortumab Vedotin	22	16.1	546.3	0.49	27	13.8	12.5	1129.0
M2	HT1376/P	Enfortumab Vedotin	22	15.6	496.9	0.54	28	14.5	9.5	685.2
M3	HT1376/P	Enfortumab Vedotin	22	14.8	424.3	0.46	27	13.2	11.9	978.7
M4	HT1376/P	Enfortumab Vedotin	22	13.8	344.0	0.37	29	12.8	12.3	1014.0
M5	HT1376-CR	Enfortumab Vedotin	22	7.3	50.9	0.10	27	6.9	5.2	97.7
M6	HT1376-CR	Enfortumab Vedotin	22	7.8	62.1	0.12	27	6.6	6.2	132.8
M7	HT1376-CR	Enfortumab Vedotin	22	10.0	130.9	0.17	30	8.8	7.8	280.3
M8	HT1376-CR	Enfortumab Vedotin	22	9.6	115.8	0.20	29	7.7	6.2	155.0

Fig. 2

Individual tumor measurements and body weight data in xenograft experiments										
Mouse ID	Tumor	Treatment	Day	Length (mm)	Tumor volume (mm3)	Tumor weight (g)	Body weight (g)	Excised tumor length (mm)	Excised tumor width (mm)	Exicised tumor volume (mm3)
M9	HT1376/P	Vehicle	1	10.0	130.9		27			
M10	HT1376/P	Vehicle	1	10.0	130.9		27			
M11	HT1376/P	Vehicle	1	10.0	130.9		26			
M12	HT1376-CR	Vehicle	1	10.0	130.9		30			
M13	HT1376-CR	Vehicle	1	10.0	130.9		31			
M14	HT1376-CR	Vehicle	1	10.0	130.9		28			
M9	HT1376/P	Vehicle	8	12.5	255.5		27			
M10	HT1376/P	Vehicle	8	14.3	382.6		27			
M11	HT1376/P	Vehicle	8	12.1	231.8		24			
M12	HT1376-CR	Vehicle	8	11.8	215.0		30			
M13	HT1376-CR	Vehicle	8	11.7	209.5		32			
M14	HT1376-CR	Vehicle	8	11.3	188.8		28			
M9	HT1376/P	Vehicle	15	15.8	516.0		25			
M10	HT1376/P	Vehicle	15	20.7	1160.5		29			
M11	HT1376/P	Vehicle	15	14.5	398.9		26			
M12	HT1376-CR	Vehicle	15	13.7	336.4		30			
M13	HT1376-CR	Vehicle	15	14.4	390.7		32			
M14	HT1376-CR	Vehicle	15	13.4	314.8		29			
M9	HT1376/P	Vehicle	22	19.3	940.6	1.50	26	19	12.3	1505.1
M10	HT1376/P	Vehicle	22	22.6	1510.2	2.06	28	21.5	14.1	2238.1
M11	HT1376/P	Vehicle	22	17.3	677.4	1.38	25	16.8	12.4	1352.5
M12	HT1376-CR	Vehicle	22	14.2	374.6	0.73	30	13.1	11.9	971.3
M13	HT1376-CR	Vehicle	22	14.9	432.8	0.52	31	13.1	11.1	845.1
M14	HT1376-CR	Vehicle	22	15.8	516.0	0.51	30	13.2	11.7	946.1

Fig. 3

b

Quantification of nectin-4 expression normalized to β-actin.

	Group A	Group B
	HT1376/P	HT1376-CR
1	1.0	0.142261
2	1.0	0.128742
3	1.0	0.290152

d Quantification of nectin-4 expression by flow cytometry

Group A	Group B
HT1376/P	HT1376-CR
1	0.06719618
1	0.10148360
1	0.12683035

f Quantification of EV-binding by flow cytometry

Group A	Group B
HT1376/P	HT1376-CR
1	0.17252544
1	0.15817451
1	0.16677316

Group A	Group B
HT1376/P	HT1376-CR
1	0.220999
1	0.179924
1	0.194287

Fig. 4

a

Relative cell viability following MMAE treatment, normalized to 0 nM.

X	Group A			Group B		
MMAE (log10 pM)	HT1376/P			HT1376-CR		
X	A:Y1	A:Y2	A:Y3	B:Y1	B:Y2	B:Y3
7.0	12.07068	11.23658	10.69584	2.616433	0.853732	1.377491
6.0	14.95271	12.69647	12.04585	3.036729	2.674524	2.322685
5.0	19.47735	19.24818	17.25559	3.623627	3.494258	3.326495
4.0	21.89647	22.54947	21.04286	6.751231	5.613478	4.920135
3.0	36.61025	41.35002	34.56555	15.365390	16.198250	15.460140
2.0	64.80836	67.18551	60.27906	40.090870	33.775310	33.224650
1.0	74.92782	87.74098	83.62327	53.453240	65.030980	57.118260
0.0	90.65207	96.82588	91.12531	83.199550	90.862040	85.008790
-1.0	98.16824	102.73160	92.18991	96.762590	98.553190	93.083240

d

Cell cycle distribution in HT1376/P and HT1376-CR cells treated without MMAE.

	Table format: Grouped	Group A			Group B		
		HT1376			HT1376-CR		
		A:1	A:2	A:3	B:1	B:2	B:3
1	G1	47.5	43.0	40.8	38.8	39.2	42.6
2	S	31.8	32.0	37.5	35.6	37.6	29.2
3	G2/M	18.8	24.8	20.5	25.0	22.7	26.0

g

Cell cycle distribution in HT1376/P and HT1376-CR cells treated with MMAE.

	Table format: Grouped	Group A			Group B		
		HT1376			HT1376-CR		
		A:1	A:2	A:3	B:1	B:2	B:3
1	G1	38.7	35.1	42.8	27.8	28.7	34.0
2	S	28.0	32.4	29.8	35.5	33.3	32.3
3	G2/M	26.3	25.6	27.3	33.6	34.7	31.9

Fig. 5

a

Quantitative PCR data showing relative expression of ABC transporter genes in HT1376/P and HT1376-CR cells.

Table format: Grouped		Group A HT1376			Group B HT1376-CR		
		A:1	A:2	A:3	B:1	B:2	B:3
1	ABCB1	1	1	1	57.303540	52.839820	70.447260
2	ABCC1	1	1	1	0.764616	0.727757	0.708498
3	ABCC2	1	1	1	3.535242	2.763076	3.606578
4	ABCG2	1	1	1	1.283040	1.165259	1.282910

Fig. 5

Comparison of relative cell number, normalized to the cell number at 0 nM, between control and ABCB1 knockdown cell

d CDDP

X	Group A			Group B			Group C		
CDDP (log2 μM)	ABCB1 KD1			ABCB1 KD2			Control		
X	A:Y1	A:Y2	A:Y3	B:Y1	B:Y2	B:Y3	C:Y1	C:Y2	C:Y3
7	0.52613568	5.883905	3.731343	0.473469	2.327515	1.579456	0.332194	1.403874	1.323799
6	2.83172213	7.519789	3.482587	1.330612	3.006373	2.331578	1.082825	2.411283	2.174812
5	3.36213534	9.604222	6.490276	2.538776	4.447215	4.147416	4.337688	5.953464	6.701732
4	15.51030880	18.284960	19.674360	12.661220	13.771130	12.173630	22.320890	23.878850	23.402870
3	35.23825820	42.348280	41.745820	40.024490	31.324470	35.618350	53.061620	56.375930	53.997990
2	67.71751220	59.208440	61.931250	68.293880	49.431980	60.997100	77.350110	84.368910	79.492940
1	86.79100010	66.253300	90.773410	80.620410	50.734280	62.619530	94.151470	87.833100	86.921580
0	97.66019330	79.182060	114.043400	89.689800	80.673320	81.530030	96.722780	101.982300	104.964200
-1	98.63974680	79.050130	123.767500	93.918370	90.911610	91.189430	105.280000	105.219000	101.749300

e MMAE

X	Group A			Group B			Group C		
MMAE (log10 pM)	ABCB1 KD1			ABCB1 KD2			Control		
X	A:Y1	A:Y2	A:Y3	B:Y1	B:Y2	B:Y3	C:Y1	C:Y2	C:Y3
7	3.815800	4.181200	2.708300	4.147900	3.712900	1.290300	2.371400	1.991200	2.395300
6	4.420900	4.645200	3.317600	4.195800	3.100000	2.304700	2.608200	2.300600	2.481400
5	4.231200	5.466300	4.628000	4.023400	5.542700	1.477100	3.290300	3.271600	2.836900
4	5.003400	6.871900	4.465200	5.546500	5.160800	5.392400	5.297800	4.867900	4.431300
3	7.685700	11.744800	6.697800	8.832300	11.955900	6.326700	16.292600	14.498200	14.435400
2	27.225100	30.379300	19.717900	28.412700	24.391500	23.384900	51.415400	49.712000	46.416400
1	67.324500	83.141500	67.262000	69.824700	67.134500	84.757100	87.626800	87.186900	108.638000
0	94.897300	114.087500	97.312500	96.426900	106.466500	101.913200	96.656700	102.047200	106.871500
-1	92.598800	111.053100	100.250400	99.118700	108.953600	102.278000	96.020500	109.521800	116.460200

f EV

X	Group A			Group B			Group C		
EV (log2 nM)	ABCB1KD1			ABCB1KD2			control		
X	A:Y1	A:Y2	A:Y3	B:Y1	B:Y2	B:Y3	C:Y1	C:Y2	C:Y3
9.718088	1.080490	1.776111	0.799526	0.586070	0.846105	0.798749	1.130397	1.375034	1.535088
8.718088	2.147857	3.086420	2.017852	1.911875	2.458493	1.918704	4.583359	4.634886	3.420067
7.718088	4.052931	5.290464	4.437582	4.649251	4.904215	4.403070	9.892520	10.303110	8.599448
6.718088	8.180227	10.876320	9.771987	9.577243	10.488510	9.346219	21.026620	24.205020	22.393060
5.718088	22.812770	14.485340	21.092260	24.161100	24.434870	20.756110	45.123230	43.455500	42.280210
4.718088	46.636050	39.604640	41.528830	45.606260	52.723340	42.637860	69.763420	70.151560	71.456730
3.718088	81.076120	79.906080	67.650920	83.493550	81.976370	69.704380	79.955530	85.062000	73.479700
2.718088	90.218720	97.481630	89.310040	99.499700	86.538950	89.218310	89.987030	87.850650	93.736450
1.718088	96.316710	113.212900	90.710270	95.332880	92.123240	86.762360	94.490090	91.843480	89.461360
