# Supplementary material for: Enhanced enfortumab vedotin sensitivity in a cisplatin-resistant bladder cancer model
Source: PLoS One. 2026 Oct 29;21(10):e0361593. doi: 10.1371/journal.pone.0361593 (PMC13718962; doi:10.1371/journal.pone.0361593)

Raw images of immunoblotting.

Original immunoblot images corresponding to Fig. 3a (nectin-4), Fig. 5b (ABCB1), and Fig. 5c (ABCB1 knockdown) are presented. The sample identities and loading order (from left to right) are indicated below each image, and the target proteins and β-actin are labeled above. Molecular weight markers are shown alongside the blots. Protein bands were detected using an enhanced chemiluminescence reagent (Cytiva, RPN2232), and images were acquired using an Amersham Imager 600 system (Cytiva). For Fig. 3a and Fig. 5b, the loading order was HT1376/P followed by HT1376-CR. For Fig. 5c, the loading order was ABCB1 KD1 followed by Control, and ABCB1 KD2 followed by Control.

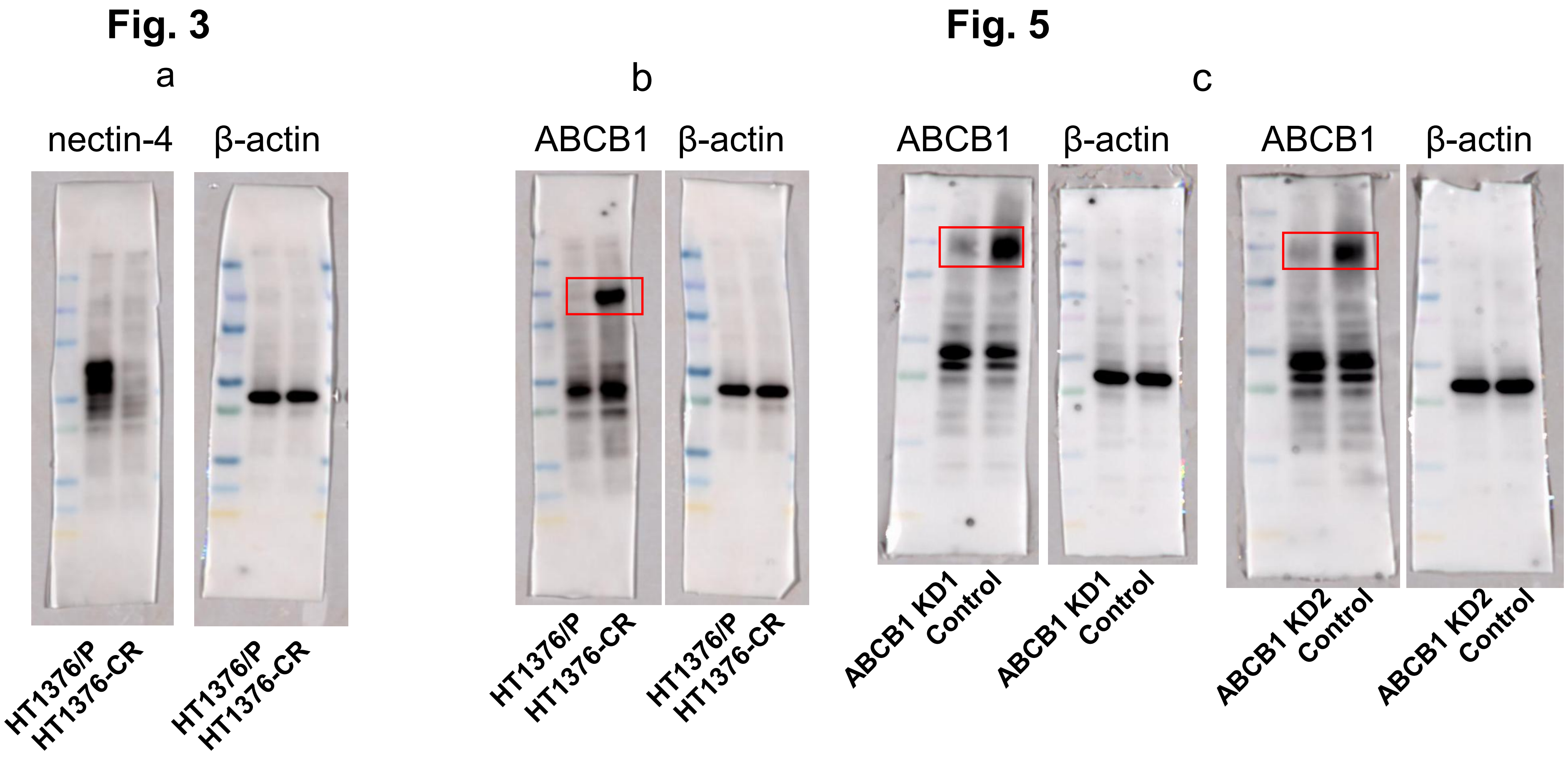

Supplement: Supplementary file 3 [file pone.0361593.s003.pdf]
